# Rapid homeostatic downregulation of LTP by extrasynaptic GluN2B receptors

**DOI:** 10.1152/jn.00421.2018

**Published:** 2018-08-15

**Authors:** Jary Y. Delgado, Ann E. Fink, Seth G. N. Grant, Thomas J. O'Dell, Patricio Opazo

**Affiliations:** ^1^Department of Physiology, University of California, Los Angeles, Los Angeles, California; ^2^Genes to Cognition Programme, Centre for Clinical Brain Science, Edinburgh University, Edinburgh, United Kingdom

**Keywords:** extrasynaptic, GluN2B, homeostasis, SAP102, scaling

## Abstract

Although the activation of extrasynaptic GluN2B-containing *N*-methyl-d-aspartate (NMDA) receptors has been implicated in neurodegenerative diseases, such as Alzheimer’s and Huntington’s disease, their physiological function remains unknown. In this study, we found that extrasynaptic GluN2B receptors play a homeostatic role by antagonizing long-term potentiation (LTP) induction under conditions of prolonged synaptic stimulation. In particular, we have previously found that brief theta-pulse stimulation (5 Hz for 30 s) triggers robust LTP, whereas longer stimulation times (5 Hz for 3 min) have no effect on basal synaptic transmission in the hippocampal CA1 region. Here, we show that prolonged stimulation blocked LTP by activating extrasynaptic GluN2B receptors via glutamate spillover. In addition, we found that this homeostatic mechanism was absent in slices from the SAP102 knockout, providing evidence for a functional coupling between extrasynaptic GluN2B and the SAP102 scaffold protein. In conclusion, we uncovered a rapid homeostatic mechanism that antagonizes LTP induction via the activation of extrasynaptic GluN2B-containing NMDA receptors.

**NEW & NOTEWORTHY** Although long-term potentiation (LTP) is an attractive model for memory storage, it tends to destabilize neuronal circuits because it drives synapses toward a maximum value. Unless opposed by homeostatic mechanisms operating through negative feedback rules, cumulative LTP could render synapses unable to encode additional information. In this study, we uncovered a rapid homeostatic mechanism that antagonizes LTP induction under conditions of prolonged synaptic stimulation via the activation of an extrasynaptic GluN2B-SAP102 complex.

## INTRODUCTION

Extrasynaptic GluN2B-containing *N*-methyl-d-aspartate (NMDA) receptors have been implicated in neurodegenerative diseases, such as Alzheimer’s and Huntington’s disease via the activation of proapoptotic signaling cascades (Hardingham and Bading 2010; Li et al. 2011). Although consistently involved in disease, the physiological function of extrasynaptic GluN2B receptors remains unknown.

In general, GluN2B-containing NMDA receptors have been implicated in various, and sometimes opposite, forms of synaptic plasticity. For example, GluN2B receptors have been implicated in the induction of long-term potentiation (LTP) and long-term depression (LTD) (Barria and Malinow 2005; Liu et al. 2004). This paradox has raised the question as to how GluN2B receptors can couple to divergent signaling pathways. Because GluN2B can interact with multiple members of the MAGUK family of scaffolding proteins, including PSD95, PSD93, and SAP102 (Grant and O’Dell 2001; Kim and Sheng 2004), and even form distinct supramolecular complexes depending on the bound MAGUK member (GluN2B receptors form 1.5-MDa supercomplexes with PSD95 and 350-kDa complexes with SAP102) (Frank and Grant 2017; Frank et al. 2016), it is plausible that different GluN2B-MAGUK supercomplexes could potentially couple to specific signaling pathways. The complexity of the GluN2B-MAGUK signaling is further increased by the preferential localization of different MAGUKs to synaptic or extrasynaptic sites. For instance, PSD95 is synaptically located at discrete nanodomains (MacGillavry et al. 2013; Nair et al. 2013), whereas SAP102 is preferentially located at extrasynaptic sites (Chen et al. 2012; Zheng et al. 2011).

Although the physiological function of extrasynaptic receptors remain unknown, it is interesting to note that their activation, not only antagonizes, but also dominates over synaptic signaling (Hardingham et al. 2002). For instance, although synaptic NMDA receptors promote ERK and cAMP response element-binding protein (CREB) phosphorylation, extrasynaptic NMDA receptors promote an ERK and CREB dephosphorylation pathway (Hardingham et al. 2002). In this context, it is possible that extrasynaptic NMDA receptors homeostatically prevent the overactivation of synaptic signaling in conditions of strong synaptic stimulation that lead to glutamate spillover.

We have previously observed that LTP in the hippocampal CA1 region can be induced by brief period of theta-pulse stimulation (TPS) (5 Hz for 15–30 s). Surprisingly, prolonging the time of stimulation (5 Hz for 1–3 min) results in no significant potentiation of synaptic transmission relative to baseline recordings (Thomas et al. 1996). In this study, we explore whether extrasynaptic GluN2B receptors might homeostatically antagonize the signaling pathways underlying the induction of LTP during periods of prolonged synaptic stimulation as previously hypothesized (Rusakov et al. 2004). Here, we show that prolonged synaptic stimulation promotes the spillover of glutamate to extrasynaptic sites, the activation of a GluN2B-SAP102 functional complex, and the homeostatic downregulation of LTP.

## MATERIAL AND METHODS

Standard techniques were used to prepare slices (400 μm thick) from the hippocampus of halothane-anesthetized 5- to 8-wk-old male C57BL/6 mice or from 4- to 7-mo-old male SAP102 knockout (KO) mice and their wild-type (WT) littermates (MF1 strain) as before (Cuthbert et al. 2007). All techniques were approved by the UCLA Institutional Animal Care and Use Committee and done in accordance with UK Home Office Guidelines.

Slices were maintained in interface-type chambers (Fine Science Tools, Foster City, CA) that were continuously perfused (at 2–3 ml/min) with a warm (30°C–31°C), oxygenated (95% O_2_-5% CO_2_) artificial cerebrospinal fluid (aCSF) containing the following: 124.0 mM NaCl, 4.4 mM KCl, 25.0 mM Na_2_HCO_3_, 1.0 mM NaH_2_PO_4_, 1.2 mM MgSO_4_, 2.0 mM CaCl_2_, and 10.0 mM glucose. In our initial experiments, field excitatory postsynaptic potentials (fEPSPs) were recorded in slices maintained in an interface-type recording chamber, whereas in later experiments extracellular recordings were done using slices completely submerged in aCSF. The type of recording configuration, which is noted in the figure legends, had no apparent effect on our observations.

Standard extracellular recording techniques were used to record fEPSPs in the CA1 region of hippocampal slices (Babiec et al. 2017). Presynaptic fiber stimulation pulses were delivered once every 50 s (0.02 Hz, 0.01- to 0.02-ms-duration pulses using tungsten wire bipolar stimulation electrodes placed in stratum radiatum of the CA1 region) using a stimulation strength that evoked fEPSPs that were approximately half of the maximal fEPSP amplitude that could be evoked by strong-intensity stimulation. LTP was induced using either short-duration trains of TPS (150 pulses of presynaptic stimulation delivered at 5 Hz) or long-duration trains of TPS (900 pulses) either in the absence (controls) or presence of inhibitors in a randomized manner to minimize bias when allocating slices to treatment. LTD and depotentiation were induced using a classical protocol at 1-Hz stimulation for 15 min (900 pulses). The average of fEPSP slopes recorded between 40 and 45 min after TPS was used for statistical comparisons. In all experiments, individual slices were considered the experimental and analytical units (*n*). The number of animals used in each experiment is stated in the corresponding figure legend (e.g., *n* = 6 from 4 animals).

Statistical significance of results was assessed using ANOVAs (followed by Bonferroni *t*-tests) or paired and unpaired *t*-tests (2-tailed). When used in reporting statistical results, asterisks denote the following significance level: ***P < 0.001. Nonstatistical significance (*P* > 0.05) is denoted by ns.

## RESULTS

**Fig. 1.**
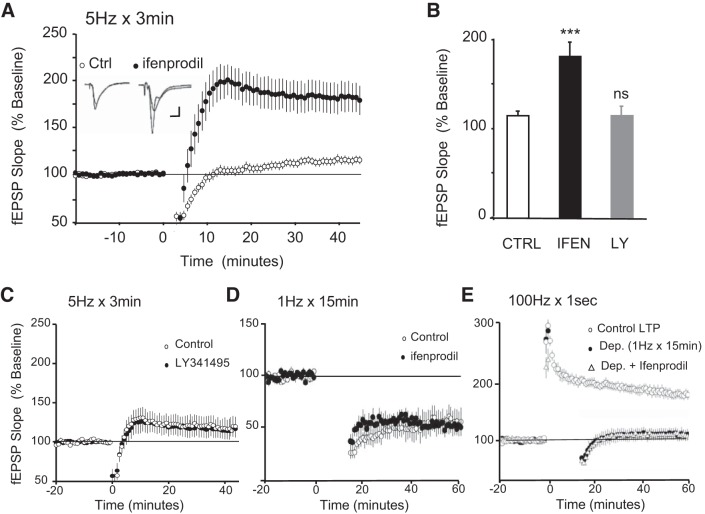
Extrasynaptic GluN2B-containing *N*-methyl-d-aspartate (NMDA) receptors homeostatically antagonize long-term potentiation (LTP). *A*: in control experiments, 3 min of 5-Hz stimulation (delivered at time = 0, ○, *n* = 14 from 12 animals) produced a small enhancement of synaptic transmission [field excitatory postsynaptic potentials (fEPSPs) were 115 ± 4% of baseline 45 min after 5-Hz stimulation). However, 5 Hz for 3 min delivered in the presence of the GluN2B blocker ifenprodil (5 μM) induced robust LTP (●, fEPSPs were 182 ± 16% of baseline 45 min after 5-Hz stimulation, *n* = 9 from 8 animals, *P* < 0.001 compared with controls). *Inset*: fEPSPs recorded during baseline and 45 min after 5-Hz stimulation in control (*left*) and ifenprodil (*right*). Calibration: 2 mV, 2.5 ms. *B*: summary of the data presented in *A* and *C*. Bar graph indicating the mean fEPSP averaged from the last 5 min of experiment in each condition [***P < 0.001 and nonstatistical significance (ns), *P* > 0.05 compared with untreated controls]. IFEN, ifenprodil; LY, LY341495. *C*: extrasynaptic metabotropic glutamate receptors do not contribute to the homeostatic pathway. A 10-min bath application of 10 μM mGluR antagonist LY341495 does not rescue LTP. fEPSPs were 113.0% of baseline for control experiments (*n* = 6 from 5 animals) and 115.1% of baseline for the experiment carried out in the presence of LY341495 (*n* = 5 from 4 animals); *P* = 0.9. *D*: GluN2B-containing NMDA receptors are not required for long-term depression (LTD). fEPSPs were 53.1% (*n* = 6 from 5 animals) and 46.7% (*n* = 5 from 4 animals) of baseline values at 60 min after LTD induction in the absence or presence of the ifenprodil 5 μM, respectively; *P* = 0.73. *E*: GluN2B-containing NMDA receptors are not required for depotentiation (1 Hz for 15 min delivered 2 min after a high-frequency LTP protocol; 2 times 100 Hz for 1 s). fEPSPs were 109.5% and 108.3% of baseline values at 60 min after depotentiation in the absence (*n* = 6 from 4 animals) or presence (*n* = 6 from 4 animals) of the ifenprodil 5 μM, respectively; *P* = 0.9.

We have previously found that the duration of TPS (5 Hz) has an important impact on the direction of synaptic plasticity (Thomas et al. 1996). In particular, brief trains of stimulation trigger LTP (5 Hz for 30 s), whereas longer stimulation times (5 Hz for 3 min) have no effect on basal synaptic transmission. Because prolonged stimulation might promote spillover to extrasynaptic sites, we reasoned that 5 Hz for 3 min might recruit a population of extrasynaptic receptors that antagonize the induction of LTP. Because NMDA receptors containing the GluN2B are enriched at extrasynaptic sites (Stocca and Vicini 1998; Tovar and Westbrook 1999), it is possible that they are recruited upon prolonged stimulation. To test this hypothesis, we stimulated at 5 Hz for 3 min in the absence and presence of 5 µM ifenprodil, a specific GluN2B inhibitor (Williams 1993). As previously shown (Thomas et al. 1996), we found that 3 min of 5-Hz stimulation has little lasting effect on synaptic strength (Fig. 1, *A* and *B*, fEPSPs were 115 ± 4% of baseline 45 min after 5-Hz stimulation, *n* = 14). However, when the same stimulation protocol was applied in the presence of ifenprodil (5 µM), a robust LTP was revealed (Fig. 1, *A* and *B*, fEPSPs were 182 ± 16% of baseline 45 min after 5-Hz stimulation, *n* = 9, *P* < 0.001). Another group of receptors enriched at peri-/extrasynaptic sites consists of metabotropic glutamate receptors (mGluR) (Baude et al. 1993). To examine whether mGluR activation might also antagonize LTP during prolonged stimulation, we stimulated at 5 Hz for 3 min in the presence of the mGluR antagonist LY341495 (10 μM). As shown in Fig. 1, *B* and *C*, LY341495 had no effect on the outcome of 5-Hz 3-min stimulation (fEPSPs were 113.0 ± 11.0% and 115.1 ± 13.0% of baseline values at 45 min after 5-Hz stimulation in the absence or presence of the mGluR antagonist, respectively, *P* = 0.90). These findings suggest that the activation of extrasynaptic GluN2B receptors but not mGluRs antagonizes the induction of LTP under prolonged theta-frequency stimulation. Although controversial, it has previously been shown that activation of GluN2B receptors can trigger LTD under certain conditions (Liu et al. 2004; but see Morishita et al. 2007). It is thus possible that 5 Hz for 3 min might trigger LTD through GluN2B receptors and thus mask the expression of LTP. To explore this alternative, we tested the contribution of GluN2B receptor signaling to a classical LTD protocol (1 Hz for 15 min). As shown in Fig. 1*D*, ifenprodil 5 µM had no effect on the induction of LTD, suggesting that extrasynaptic GluN2B seems to antagonize LTP rather than simply mask LTP via LTD induction (fEPSPs were 53.1% and 46.7% of baseline values at 60 min after LTD induction in the absence or presence of the ifenprodil 5 μM, respectively; *P* = 0.73). As for LTD and consistent with previous studies (Massey et al. 2004), we found that GluN2B receptors do not mediate depotentiation of synaptic transmission (Fig. 1*E*; 1 Hz for 15 min delivered 2 min after a high-frequency stimulation LTP protocol, 2 times 100 Hz for 1 s; fEPSPs were 109.5% and 108.3% of baseline values at 60 min after depotentiation in the absence or presence of the ifenprodil 5 μM, respectively; *P* = 0.9).

**Fig. 2.**
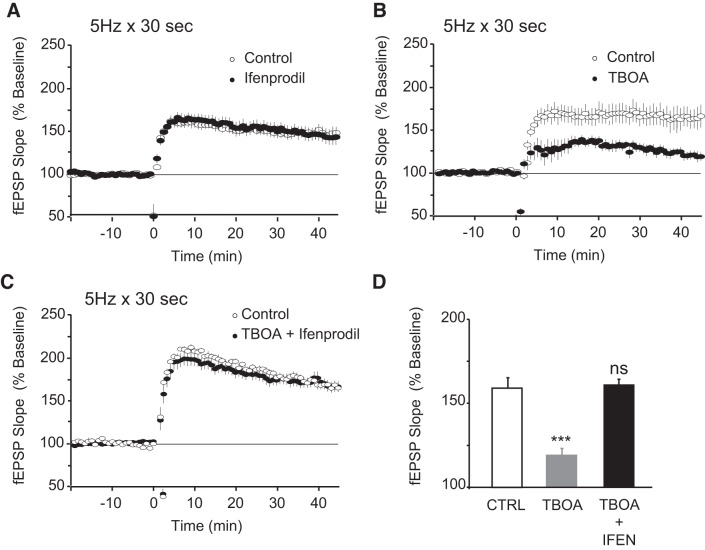
Extrasynaptic GluN2B homeostasis is recruited after glutamate spillover. *A*: 5-Hz stimulation for 30 s delivered at time = 0 induced indistinguishable robust long-term potentiation (LTP) in vehicle control experiments and in the presence of ifenprodil 5 μM. Field excitatory postsynaptic potentials (fEPSPs) were 146.7% of baseline 45 min after 5-Hz stimulation for control (○, *n* = 8 from 6 animals) and 143.7% of baseline in the presence of ifenprodil (●, *n* = 5 from 5 animals); *P* = 0.93. *B*: 5-Hz, 30-s stimulation delivered at time = 0 induced robust LTP (fEPSPs were 166.5% of baseline in vehicle control experiments; ○, *n* = 6 from 6 animals) but was significantly impaired in the presence of the glutamate transporter blocker DL-threo-beta-benzyloxyaspartic acid (TBOA) 10 μM (fEPSPs were 119.2% of baseline; ●, *n* = 6 from 5 animals; *P* < 0.001 compared with controls). *C*: 5-Hz stimulation for 30 s induced indistinguishable robust LTP in vehicle control experiments (○, *n* = 11 from 10 animals, fEPSPs were 160.4% of baseline) and when delivered in the presence of TBOA 10 μM and ifenprodil 5 μM (●, *n* = 8 from 7 animals; fEPSPs were 161.3% of baseline); *P* = 0.91. *D*: summary of the data presented in *A*–*C*. Bar graph indicates the average fEPSP obtained from the last 5 min of recording for each condition (***P < 0.001 compared with untreated controls). IFEN, ifenprodil.

We next examined whether brief stimulation at 5 Hz, which we have previously shown to induce robust LTP, might be sufficient to activate the extrasynaptic pool of GluN2B receptors. We therefore stimulated slices at 5 Hz for 30 s in the presence of ifenprodil (5 μM). As shown in Fig. 2, *A* and *D*, we found that 5 Hz for 30 s induces LTP of similar magnitude in the absence or presence of ifenprodil (fEPSPs were 143.7 ± 6.0% and 146.7 ± 11.0% of baseline 45 min after 5-Hz stimulation with or without ifenprodil, respectively, *P* = 0.930). This indicates that extrasynaptic GluN2B populations are preferentially recruited after prolonged period of stimulation and that 30 s of 5-Hz stimulation does not induce sufficient spillover to activate these receptors in a way that causes synaptic plasticity. Because glutamate uptake is the main mechanism to prevent spillover (Huang and Bergles 2004), we examined whether blocking glutamate transporters might facilitate the activation of GluN2B receptors even during 5 Hz for 30 s, as it has been previously observed with 100-Hz stimulation (Scimemi et al. 2009; Varga et al. 2015). To test this hypothesis, we stimulated at 5 Hz for 30 s in the presence and absence of the general glutamate transporter inhibitor DL-threo-beta-benzyloxyaspartic acid (TBOA). As shown in Fig. 2, *B* and *D*, LTP induced by stimulation at 5 Hz for 30 s was significantly impaired in the presence of TBOA 10 μM (fEPSPs were 166.5 ± 11.0% of baseline 45 min after 5-Hz stimulation in the absence and 119.2 ± 5.0% in the presence of TBOA, *P* < 0.001). To confirm that LTP impairment was due to activation of GluN2B-containing NMDA receptors, we applied TBOA in the presence of the GluN2B blocker ifenprodil 5 μM. As shown in Fig. 2, *C* and *D*, ifenprodil completely rescued the LTP impairment caused by TBOA 10 μM (fEPSPs were 160.4 ± 5.0% and 161.3 ± 6% of baseline at 45 min after 5-Hz stimulation for control and in the presence of TBOA and ifenprodil, respectively, *P* = 0.91). Taken together, these findings support the hypothesis that extrasynaptic GluN2B receptors antagonize LTP in conditions that promote glutamate spillover, such as prolonged stimulation or glutamate transporter block.

**Fig. 3.**
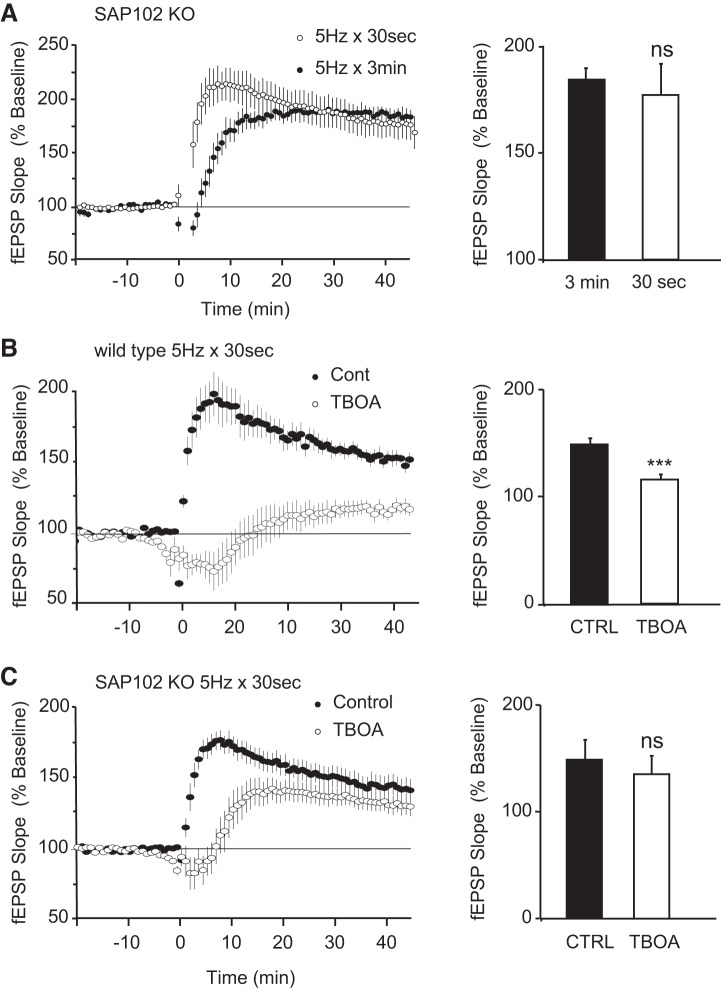
Extrasynaptic GluN2B homeostasis is uncoupled in the SAP102 knockout (KO) mice. *A*, *left*: indistinguishable amount of long-term potentiation (LTP) is induced by both short (30 s) and long (3 min) periods of 5-Hz stimulation in the SAP102 KO mice. Field excitatory postsynaptic potentials (fEPSPs) were 177% (*n* = 11 from 5 animals) and 184% (*n* = 10 from 6 animals) of baseline for 30 s and 3 min, respectively. *Right*: bar graph indicating the average fEPSP obtained from the last 10 min of recording for each condition (*P* = 0.12). *B*, *left*: 5-Hz, 30-s stimulation delivered at time = 0 induced robust LTP in wild-type littermates. fEPSPs were 159.5% of baseline in vehicle control experiments (●, *n* = 6 from 4 animals) but were significantly impaired in the presence of the glutamate transporter blocker DL-threo-beta-benzyloxyaspartic acid (TBOA) (fEPSPs were 116.7% of baseline; ○, *n* = 6 from 4 animals). *Right:* bar graph indicating the average fEPSP obtained from the last 10 min of recording for each condition (***P < 0.001). *C*, *left*: 5-Hz stimulation for 30 s caused a strong potentiation in the SAP102 KO mice either in the presence or absence of TBOA. fEPSPs were 149.6% (*n* = 6 from 4 animals) and 141.7% (*n* = 8 from 4 animals) of baseline in the absence or presence of TBOA, respectively. *Right*: bar graph indicating the average fEPSP obtained from the last 5 min of recording for each condition. *P* = 0.279.

The involvement of GluN2B receptors in various, and sometimes opposite, forms of synaptic plasticity raises the question of how GluN2B can activate divergent signaling cascades. Numerous studies have stressed the central role of MAGUK family scaffolding proteins in coupling NMDA receptors to specific signaling pathways (Grant and O’Dell 2001; Kim and Sheng 2004). Because GluN2B preferentially interacts with the extrasynaptically enriched SAP102 scaffolding protein (Chen et al. 2011; Murata and Constantine-Paton 2013; Sans et al. 2000; Zhang and Diamond 2009), it is likely that a GluN2B-SAP102 complex mediates signaling at extrasynaptic sites. To test this hypothesis, we stimulated hippocampal slices obtained from SAP102 KO mice with short and long trains of 5-Hz stimulation. Given the absence of SAP102, we hypothesized that activation of extrasynaptic GluN2B might be uncoupled from the signaling cascades that antagonize LTP. Consistent with this idea and our previous work (Cuthbert et al. 2007), we found that prolonged stimulation (5 Hz for 3 min) triggered an LTP as robust as that induced with brief stimulation (5 Hz for 30 s) (Fig. 3*A*; fEPSPs were 177% and 184% of baseline for 5 Hz for 30 s and 5 Hz for 3 min, respectively; *P* = 0.12). To make sure that this finding was not simply attributable to a deficit in synaptic transmission and subsequent failure to activate extrasynaptic GluN2B receptors, we promoted glutamate spillover using the glutamate transporter inhibitor TBOA. Although TBOA blocked LTP induced by stimulation at 5 Hz for 30 s in WT littermates (Fig. 3*B*), it caused no significant impairment in SAP102 mutant mice (Fig. 3*C*). In WT, fEPSPs were 151.5 ± 4.9% and 116.7 ± 4.7% of baseline levels in the absence or presence of TBOA, respectively (*P* < 0.001). In SAP102 KO, fEPSPs were 149.2 ± 11.0% and 141.7 ± 6.0% of baseline levels in the absence or presence of TBOA, respectively (*P* = 0.279). Taken together, these findings suggest that activation of an extrasynaptic GluN2B-SAP102 functional complex during prolonged theta-pulse stimulation activates signaling cascades that antagonize the induction of LTP.

## DISCUSSION

Although LTP is an attractive model for memory storage, it tends to destabilize neuronal circuits because it drives synapses toward a maximum value (Abbott and Nelson 2000). Unless opposed by homeostatic mechanisms operating through negative feedback rules, cumulative LTP could render synapses unable to encode additional information. In this study, we describe a novel homeostatic mechanism that rapidly antagonizes LTP induction when the synapse is faced with prolonged durations of synaptic stimulation. We have previously found that brief periods of theta-pulse stimulation (5 Hz for 15–30 s) trigger LTP in the CA1 region of the hippocampus, whereas increasing the period of stimulation (5 Hz for 1–3 min) has no lasting effects on basal synaptic transmission (Thomas et al. 1996). In this study, we found that prolonging stimulation times promotes glutamate spillover to extrasynaptic sites and recruitment of a GluN2B-SAP102 functional complex that antagonizes LTP induction.

A number of additional homeostatic mechanisms might compensate for activity-dependent increases in excitatory synaptic drive, the most studied being synaptic scaling (Turrigiano et al. 1998). To compensate for increases in activity, synaptic scaling operates through negative feedback rules by globally scaling down the strength of all synapses in a neuron (Turrigiano et al. 1998). Although synaptic scaling seems an ideal mechanism to compensate for the increased excitatory drive caused by LTP, it has the disadvantage of being slow (requiring hours and days of altered activity to take place) and nonspecific (it modifies all synapses in a neuron). Because LTP can be induced very rapidly (standard LTP protocols take a few seconds), synapses can reach a maximum value very quickly, rendering the synapse unable to encode additional information. The disparity in the time courses of homeostatic scaling and LTP outlines a need for a fast downregulating homeostatic mechanism (Keck et al. 2017; Zenke et al. 2013), such as the extrasynaptic GluN2B-mediated mechanism described in this study.

This study also emphasizes the critical role of glutamate transporters in regulating synaptic transmission and plasticity through activation of extrasynaptic GluN2B receptors. We found that even brief trains of stimulation can activate these receptors under pharmacological blockade of glutamate transporters, either directly via glutamate spillover or indirectly via some other mechanism. These results are consistent with the LTP deficits found in mice lacking either the glial (GLT-1) or neuronal glutamate transporter (EAAC1) (Katagiri et al. 2001; Scimemi et al. 2009). Interestingly, the LTP deficits observed in the EAAC1^−/−^ mice can be rescued with the GluN2B antagonist ifenprodil (Scimemi et al. 2009).

Although most GluN2B-containing NMDA receptors are located extrasynaptically, they can also be found at synaptic sites (Thomas et al. 2006). Because of this, we cannot completely rule out the possibility that synaptic GluN2B-containing NMDA receptors also contribute to the homeostatic process. Nevertheless, this possibility is unlikely because the GluN2B blocker ifenprodil does not further increase the amount of LTP obtained with brief trains of stimulation (5 Hz for 30 s). It is also unlikely that ifenprodil failure to enhance LTP is attributable to saturation of synaptic potentiation because we have previously shown that high-frequency stimulation (100 Hz for 1 s) can increase the amount of LTP obtained with this pattern of stimulation (5 Hz for 30 s) (Moody et al. 1999). Furthermore, the fact that even a brief period of stimulation can recruit the GluN2B-mediated homeostatic pathway when delivered in the presence of the glutamate transporter blocker strongly suggests that only extrasynaptic GluN2B can antagonize LTP.

Given that the GluN2B-mediated homeostatic mechanism was absent in SAP102 KO mice, our study suggests that extrasynaptic GluN2B acts in a complex with the scaffolding protein SAP102 to antagonize LTP induction. Although the present finding could be explained by a lack of GluN2B in the SAP102 KO, we have previously shown that GluN2B location and function are intact in this mutant (Cuthbert et al. 2007). The functional coupling between extrasynaptic GluN2B and SAP102 is consistent with their preferential binding (Chen et al. 2011; Murata and Constantine-Paton 2013; Sans et al. 2000) and extrasynaptic location (Petralia 2012; Zheng et al. 2011).

The potential relevance of this novel extrasynaptic GluN2B/SAP102-mediated homeostatic mechanism in vivo has been recently highlighted in a study showing that loss of the extrasynaptic GluN2B-SAP102 complex is associated with seizure susceptibility (Okuda et al. 2017). On the other hand, it has been shown that overactivation of extrasynaptic GluN2B attributable to alterations in glutamate uptake underlies the LTP deficits found in Alzheimer’s disease models (Li et al. 2011; Varga et al. 2015). In fact, it has been shown that promoting glutamate spillover with TBOA mimics and occludes the LTP deficits mediated by oligomeric Aβ (Huang et al. 2018; Li et al. 2009, 2011; Varga et al. 2015). In light of our present findings, it is plausible that the aberrant activation of the extrasynaptic GluN2B-SAP102 homeostatic mechanism described here might contribute to the LTP deficits found in Alzheimer’s disease models.

In conclusion, we have uncovered a novel homeostatic mechanism that opposes LTP induction under conditions of prolonged theta-pulse stimulation at excitatory synapses in the hippocampal CA1 region. Because this feedback loop is built into the synapse and its surrounding extrasynaptic site, it is fast and presumably input specific and thus complements other homeostatic mechanisms operating at slower time scales such as scaling (Zenke et al. 2013).

## GRANTS

This work was supported by HHS, NIH, National Institute of Mental Health (NIMH) (T. O'Dell), Welcome Trust Grant 202932 (S. Grant), and the European Research Council under the European Union Horizon 2020 research and innovation program (Grant Agreement 695568) (S. Grant).

## DISCLOSURES

No conflicts of interest, financial or otherwise, are declared by the authors.

## AUTHOR CONTRIBUTIONS

J.Y.D., T.J.O., and P.O. conceived and designed research; J.Y.D., A.E.F., and P.O. performed experiments; J.Y.D., A.E.F., and P.O. analyzed data; J.Y.D., A.E.F., and P.O. interpreted results of experiments; P.O. prepared figures; P.O. drafted manuscript; J.Y.D., A.E.F., S.G.G., T.J.O., and P.O. edited and revised manuscript; J.Y.D., A.E.F., S.G.G., T.J.O., and P.O. approved final version of manuscript.
